# Equine parvovirus hepatitis

**DOI:** 10.1111/evj.13477

**Published:** 2021-07-04

**Authors:** Anna Sophie Ramsauer, Marcha Badenhorst, Jessika‐M. V. Cavalleri

**Affiliations:** ^1^ Department for Companion Animals and Horses University Equine Clinic – Internal Medicine University of Veterinary Medicine Vienna Vienna Austria

**Keywords:** EqPV‐H, horse, liver, serum hepatitis, Theiler´s disease, *Ungulate copiparvovirus 6*

## Abstract

Equine parvovirus hepatitis (EqPV‐H) was first described in 2018 in a fatal case of Theiler's disease which followed the administration of an equine‐origin biological product. The virus has since been frequently identified in serum and liver tissue of horses affected by Theiler's disease—an acute, severe hepatitis characterised by fulminant hepatic necrosis with a fatal outcome in most cases. EqPV‐H is hepatotropic, appears to be associated with subclinical to severe hepatitis in horses, and is a likely cause of Theiler's disease. Although this disease is most frequently reported following the administration of equine‐origin biological products, it can also occur among in‐contact horses. Horizontal transmission may be iatrogenic, via contaminated equine‐origin biological products such as equine serum, botulism or tetanus antitoxin, and mesenchymal stem cells or by means of the oral route of infection. Other horizontal transmission routes, for example, arthropod vectors, warrant further investigation. A worldwide prevalence of EqPV‐H antibodies and DNA has been reported in asymptomatic horses. EqPV‐H‐positive horses suffering from acute, severe hepatitis have reportedly developed clinical signs including icterus, lethargy, inappetence, and neurological abnormalities and have had increased liver‐associated biochemistry parameters recorded. The most common histopathological abnormalities of the liver have been hepatocellular necrosis, collapse of the lobular architecture, and lymphocytic infiltration. Most horses infected experimentally with EqPV‐H have developed subclinical hepatitis, and close temporal associations between peak viraemia, seroconversion, and the onset of hepatitis have been observed. Based on strong evidence indicating that EqPV‐H causes hepatitis in horses, veterinarians should consider this virus an important differential diagnosis in such cases. Potential risks associated with the administration of equine‐origin biological products must be emphasised.

## 1 EQUINE ACUTE HEPATITIS

Theiler's disease, an acute hepatitis frequently occurring several weeks after administration of a biological substance of equine origin, has also been termed serum hepatitis, acute hepatic necrosis, or serum‐associated hepatitis.^1^ The aetiology of Theiler's disease, the most common cause of acute, severe hepatitis and liver failure in horses, has remained unidentified for more than a century.^2^ Sir Arnold Theiler described this disease for the first time in 1918 in South Africa, when approximately 2% of horses developed acute hepatic necrosis and fulminant liver failure several weeks after the administration of convalescent equine antiserum.^3^ Since then, the condition of equine acute hepatitis has been reported worldwide in association with the administration of various equine‐origin biological products, including equine plasma, tetanus antitoxin, botulism antitoxin, antiserum against *Streptococcus equi*, and allogenic stem cells.^4^, ^5^, ^6^, ^7^, ^8^, ^9^, ^10^, ^11^, ^12^, ^13^, ^14^ The condition has also been reported in horses with no history of receiving biological products and in horses that were in contact with cases of acute hepatitis.^3^, ^14^, ^15^ Due to the infectious and contagious nature of the disease, viral aetiology was suspected despite no viral agent being successfully cultured from affected animals.^2^ Due to advances in unbiased deep sequencing (next generation sequencing [NGS]) during recent years, four novel equine viruses, including two pegiviruses (Theiler's disease associated virus/pegivirus D/TDAV and equine pegivirus/pegivirus E/EPgV), a hepacivirus (equine hepacivirus/EqHV), and a parvovirus (equine parvovirus hepatitis/EqPV‐H) were identified as potential aetiological agents in samples from horses.^2^, ^13^, ^16^, ^17^, ^18^ Follow‐up investigations shed more light on the pathogenicity of these viruses. TDAV was identified as a possible cause of Theiler's disease in 2013,^13^ and it was later shown that all TDAV‐positive horses in the original study were coinfected with EqPV‐H.^16^ For both equine pegiviruses, it was recently demonstrated that they are not hepatotropic and not associated with hepatitis but instead cause persistent infection of bone marrow.^19^ In contrast, EqHV and EqPV‐H are hepatotropic and have frequently been detected in cases of mild or subclinical hepatitis.^2^, ^20^, ^21^ EqHV infection has only been reported in three cases of severe clinical liver disease.^22^, ^23^, ^24^ EqHV was associated with chronic, severe hepatopathy in two cases^23^, ^24^ and detected in one case of acute severe hepatitis.^22^ However, two of these cases were published before the detection of EqPV‐H, and no EqPV‐H testing was performed.^22^, ^24^ Therefore, EqPV‐H infection could be excluded in only one case of chronic severe hepatopathy.^23^ Since the discovery of EqPV‐H and with the exception of a single case,^15^ EqPV‐H has been detected in all recently reported cases of Theiler's disease.^14^, ^15^, ^16^, ^25^, ^26^, ^27^ EqPV‐H is, therefore, a likely cause of subclinical to severe hepatitis and presumably the causative agent of Theiler's disease.^2^ This review focuses on the current knowledge pertaining to EqPV‐H. The chronological order of publication of research studies and case reports pertaining to EqPV‐H is illustrated in Table 1.

**TABLE 1:** Chronological order of EqPV‐H‐related publications

Year of publication	Country	Study description	Study population	Reference
2013	USA	Outbreak investigation (TDAV) Epidemiological surveillance (TDAV) Experimental inoculation (TDAV) Viral genetic analysis (TDAV)	Horses, n = 22 (Farm A) Horses, n = 60 (Farms A, B, and D) Horses, n = 4	[13]
2018	USA	Case report Experimental inoculation Epidemiological surveillance Viral genetic analysis	Horse, n = 1 Horses, n = 2 Horses, n = 100	[16]
	USA	Case report	Horse, n = 1	[27]
	China	Epidemiological surveillance Viral genetic analysis	Horses, n = 143	[32]
	USA	Clinical case series	Horses, n = 18	[14]
	USA	Clinical case series Epidemiological surveillance	Horses, n = 10 Horses, n = 37	[15]
2019	New Zealand, USA, Italy, Germany, and Canada	Epidemiological surveillance Viral genetic analysis	Equine serum pools, n = 18	[37]
	USA	Metagenomic viral surveillance Epidemiological surveillance	Horses, n = 27 (clinically abnormal) Horses, n = 41 (clinically normal)	[34]
	Germany	Epidemiological surveillance Viral genetic analysis	Horses, n = 392	[35]
2020	Canada	Outbreak investigation Viral genetic analysis	Horses, n = 60 (approximately)	[25]
	Slovenia	Clinical case series	Horses, n = 4	[26]
	China	Epidemiological surveillance Viral genetic analysis	Horses, n = 60	[36]
	USA	Experimental inoculation Vertical transmission investigation	Horses, n = 17 Mares, n = 24; foals, n = 24	[21]
2021	Austria	Epidemiological surveillance Viral genetic analysis	Horses, n = 259; donkeys, n = 13	[33]

Note

This table illustrates the chronological order of publication of research studies and case reports pertaining to equine parvovirus hepatitis (EqPV‐H), since the initial report of the virus by Divers et al in February 2018. An earlier study pertaining to TDAV by Chandriani et al^13^ is included. Inclusion is based on the statement by Divers et al^16^ that retrospective testing of administered antitoxin and serum samples of recipient horses revealed the presence of EqPV‐H in all samples that tested TDAV‐positive during the 2013 investigation.

## 2 VIROLOGICAL CHARACTERISATION OF EQPV‐H

A novel equine parvovirus was identified in 2018 in the serum and liver of a horse that died of equine serum hepatitis in Nebraska, USA, 65 days after administration of tetanus antitoxin of equine origin.^16^ The administered tetanus antitoxin also contained DNA of this parvovirus.^16^ This newly identified virus belongs to the family *Parvoviridae*, subfamily *Parvovirinae*, genus *Copiparvovirus*, and species *Ungulate copiparvovirus 6* and was assigned the name equine parvovirus hepatitis (EqPV‐H).^16^, ^28^ The single‐stranded DNA virus exists as episomes in the liver, and the genome comprises 5,308 nucleotides (nt), encoding two large open reading frames (ORFs).^16^ The first ORF (1‐1779) encodes a nonstructural (NS) protein, and the second ORF (1801‐4722) encodes the structural proteins (VPs).^16^ Two intergenic regions of 21 nt and 583 nt, respectively, connect the ORFs.^16^ DNA folding software has predicted that the long intergenic region forms one long hairpin and several other small, hairpin‐like structures.^16^

Parvoviruses have a small genome that does not encode for the proteins required for viral replication.^29^ Therefore, they are thought to require the replication machinery from helper viruses or from the actively dividing cells of the host.^29^ Alternatively, parvoviruses can also activate and use the DNA damage‐repair machinery in non‐dividing cells for replication.^30^, ^31^ The mechanisms by which EqPV‐H is replicating in the liver—a typically quiescent organ with minimal dividing cells—warrant future investigation.^2^

## 3 PREVALENCE AND DISTRIBUTION OF EQPV‐H

EqPV‐H seemingly has a global distribution without apparent geographical restrictions. Surveillance studies in clinically healthy horse populations in the United States, China, Germany, and Austria have demonstrated DNA prevalence between 7.1% and 17% (Tables 2A and 2B)^16^, ^32^, ^33^, ^34^, ^35^, ^36^ and seroprevalence between 15% and 34.7% (Tables 2A and 2B).^16^, ^33^, ^35^ Markedly higher DNA prevalence has been reported on farms with recently documented cases of equine serum hepatitis in the United States and Canada (Tables 2A and 2B).^15^, ^21^, ^25^ The first report of EqPV‐H‐associated serum hepatitis in Europe came from Slovenia, where four horses on one farm were diagnosed with fatal Theiler's disease.^26^ Liver tissue from all four horses tested PCR‐positive for EqPV‐H (Table 2B).^26^ Analysis of commercial equine serum pools from New Zealand, the United States, Italy, Germany, and Canada for EqPV‐H yielded 61.1% (11/18) PCR‐positivity and 77.8% (14/18) seropositivity.^37^ All EqPV‐H DNA‐positive commercial serum samples were also positive for EqPV‐H antibodies, and three additional samples were exclusively EqPV‐H antibody‐positive.^37^ The presence of EqPV‐H DNA and antibodies in these commercial samples further supports the proposed global distribution of the virus.^37^

**TABLE 2:** EqPV‐H DNA and antibodies reported worldwide in (A) horse populations and (B) individual horses with nonexperimental EqPV‐H infection

Country	EqPV‐H DNA detected in	No. of PCR‐positive horses	No. of antibody‐positive horses	Publication reference
**A**.				
Clinically healthy horse populations				
USA	Serum	13/100 (13%)	15/100 (15%)	[16]
China	Serum	17/143 (11.9%)	n.a.	[32]
USA	Plasma	7/41 (17%)	n.a.	[34]
Germany	Serum	28/392 (7.1%)	136/392 (34.7%)	[35]
China	Serum	5/60 (8.3%)	n.a.	[36]
Austria	Serum	23/259 (8.9%)	78/259 (30.1%)	[33]
Horse populations on properties with recently documented cases of Theiler's disease				
USA	Serum	20/37 (54%)	n.a.	[15]
Canada	Serum	34/55 (61.8%)	n.a.	[25]
USA	Serum	15/24 (62.5%) mares	n.a.	[21]
USA	Serum	19/24 (79%) foals	n.a.	[21]
**B**.				
Individual horses with acute, fatal hepatitis				
USA	Serum and liver	1	n.a.	[16]
USA	Serum and/or liver	18	n.a.	[14]
USA	Serum and/or liver	9	n.a.	[15]
Canada	Serum	3	n.a.	[25]
Slovenia	Liver	4	n.a.	[26]
Individual horses with clinical, nonfatal hepatitis				
USA	Serum	4	n.a.	[13, 16]
USA	Serum and liver	1	1	[27]
Canada	Serum	1	n.a.	[25]
Individual horses with subclinical increases in liver‐associated biochemistry parameters				
USA	Serum	4	n.a.	[13, 16]
USA	Serum	7 (↑GGT)	n.a.	[15]
China	Serum	1 (↑GGT) 1 (↑ALT)	n.a.	[36]
Canada	Serum	5 (↑GGT) 4 (↑GLDH) 4 (↑BA) 7 (↑ALP) 2 (↑AST)	n.a.	[25]
Austria	Serum	2 (↑GGT)	1	[33]

Abbreviations: ALP, alkaline phosphatase; ALT, alanine aminotransferase; AST, aspartate aminotransferase; BA, bile acids; EqPV‐H, equine parvovirus hepatitis; GGT, gamma‐glutamyl transferase; GLDH, glutamate dehydrogenase; n.a., data not available; SDH, sorbitol dehydrogenase.

## 4 PHYLOGENETIC ANALYSIS

Phylogenetic analysis of the NS and VP proteins revealed that EqPV‐H shares <50% protein identity with its phylogenetic relatives in the genus *Copiparvovirus*, which infect pigs, cows, deer, sea lions, and horses.^16^, ^32^, ^34^, ^38^, ^39^ To date, two further equine copiparvoviruses, namely, equine parvovirus cerebrospinal fluid (CSF) and eqcopiparvovirus, have been identified in horses and sequenced.^34^, ^38^ Additionally, two earlier reports of equine parvoviruses exist in the literature. The first report of a hepatotropic equine parvovirus dates back to 1985, when an equine parvovirus was isolated from the liver of an aborted equine foetus, following 16 cases of abortion on a farm in Canada.^40^ Additionally, an equine parvovirus was linked to equine synovitis in Australia in 2014.^41^ As no sequence information is available for these two parvoviruses, it is unknown whether these viruses differ from the known equine parvoviruses. EqPV‐H is more closely related to porcine and bovine copiparvoviruses than to the two other equine copiparvoviruses, suggesting different evolutionary origins of the equine viruses.^16^, ^32^, ^34^, ^38^ According to the recent reorganisation of the family *Parvoviridae* and the thereby revised taxonomy based on host association, EqPV‐H was assigned to the species *Ungulate copiparvovirus 6*.^28^

Recently, all 12 publicly available complete coding sequences (CDS) of the species *Ungulate copiparvovirus 6*—obtained in China and the United States—were compared with the CDS of four Austrian variants and found to be closely related.^33^ The genetic diversity of the NS and VP genes appears to be low among different EqPV‐H variants in the United States, China, Canada, New Zealand, Italy, and Germany, indicating high conservation and low genetic variability of variants circulating globally.^16^, ^25^, ^32^, ^35^, ^37^ Eleven years after the first outbreak of Theiler's disease on a farm in Canada, the same partial NS1 sequence was obtained from a horse that died of Theiler's disease 8 weeks after arrival on the farm. This suggests that highly similar EqPV‐H variants were circulating on this farm for an extended period of time.^25^ Based on unique nucleotide substitutions in EqPV‐H variants obtained from a farm in China, distinct geographical patterns were observed.^32^ Additionally, a recent report provided evidence of genetic diversity among different strains and natural recombination between Chinese and American EqPV‐H strains within the VP1 protein.^36^ Large‐scale epidemiological studies investigating genetic diversity, recombination events, and their influence on EqPV‐H pathogenicity and clinical presentation are still lacking.

## 5 DISEASE ASSOCIATION

Since its discovery, EqPV‐H DNA has been detected in serum and/or liver of multiple patients with acute, severe clinical hepatitis, including single cases and larger outbreaks in the United States, Canada, and Slovenia.^14^, ^15^, ^16^, ^25^, ^26^, ^27^ The disease has frequently been associated with the administration of equine‐origin biological products^14^, ^16^, ^26^, ^27^ but has also been reported in patients with no history of biological product administration.^15^, ^25^, ^26^ In horses that had contact with cases of EqPV‐H‐associated acute hepatitis, the prevalence of hepatitis was high (15%–27%) and significantly associated with EqPV‐H infection.^15^, ^25^, ^26^ Although in‐contact horses can develop subclinical to severe clinical hepatitis,^15^, ^25^, ^26^ majority of infected horses appear clinically normal.^15^, ^25^, ^26^

The clinical signs most frequently observed in affected horses are icterus, lethargy, inappetence, and neurological signs such as blindness, obtundation, head pressing, and ataxia.^13^, ^14^, ^15^, ^16^, ^21^, ^25^, ^26^, ^27^ The spectrum of clinical signs described in EqPV‐H‐affected horses in various studies is summarised in Table 3A. Liver‐associated biochemistry parameters like gamma‐glutamyl transferase (GGT), aspartate aminotransferase (AST), sorbitol dehydrogenase (SDH), glutamate dehydrogenase (GLDH), alkaline phosphatase (ALP), alanine aminotransferase (ALT), bilirubin, bile acids, and ammonia are frequently increased in infected horses, whereas glucose decreased in some horses (Table 3B).^14^, ^15^, ^25^, ^26^, ^27^ The outcome in the majority of horses with severe clinical signs is fatal, ranging from sudden death to humane euthanasia within days of the appearance of clinical signs.^14^, ^15^, ^16^, ^25^, ^26^ Livers have been described as atrophic, flattened, flabby, friable, and/or discoloured upon post‐mortem examination.^14^, ^26^, ^27^ The most commonly reported histopathological abnormalities include hepatocellular necrosis, collapse of the lobular architecture, and lymphocytic infiltration.^14^, ^15^, ^16^, ^21^, ^26^, ^27^ However, majority of horses that were tested for EqPV‐H due to contact with affected horses or for prevalence studies did not show any obvious clinical signs. Nevertheless, subclinical hepatitis with elevated liver‐associated biochemistry parameters could be detected in some (Table 3B).^13^, ^15^, ^25^, ^33^, ^36^

**TABLE 3:** Clinical signs (A) and blood parameters (B) observed in EqPV‐H infected horses

Clinical signs observed in EqPV‐H affected horses	Number of horses total	Number of horses according to reference no							
		[14]	[15]	[27]	[25]	[26]	[21]	[13]	16]
**A**.									
Neurological clinical signs	25	12	10		1	3			1
Blindness	14	10	3		1				
Obtundation	13	10	3						
Head pressing	12	10	1			1			
Ataxia	10	2	4		1	3			
Recumbency	3	1	1			1			
Further clinical signs									
Icterus	25	9	5	1	2	2	1	4	1
Lethargy	18	7		1		4	1	4	1
Inappetence	14	7		1		1	1	4	
Bilirubinuria	6	5							1
Photodermatitis	5							5	
Colic	3	2				1			
Dehydration	2					2			
Pyrexia	2	2							
Bleeding diathesis	2		2						
Hyperhidrosis	1				1				
Decreased athletic performance	1			1					

Most frequently altered blood parameters	Number of horses total	Number of naturally infected horses with clinical disease (reference number)					Number of naturally subclinical infected horses (reference number)						Experimentally infected horses (reference number)			
		[14]	[15]	[25]	[27]	[26]	[13]	[32]	[15]	[25]	[36]	[33]	[16]	[21]	[13]	
**B**.																
GGT (increased)	55	13/13	9/9	3/4	1/1	4/4	*	0/17	7/20	5/31	1/5	2/23	2/2	7/10	1/4	
AST (increased)	38	12/12	8/8	2/3	1/1	3/3	*	0/17	n.a.	2/31	1/5	n.a.	2/2	6/10	1/4	
Bilirubin (increased)	33	13/13	8/8	2/3	1/1	4/4	2/15	n.a.	n.a.	0/31	n.a.	n.a.	2/2	1/10	n.a.	
Bile acids (increased)	27	6/6	3/3	3/4	1/1	n.a.	2/15	n.a.	n.a.	4/31	n.a.	n.a.	2/2	6/10	n.a.	
SDH (increased)	12	n.a.	n.a.	n.a.	0/1	n.a.	*	n.a.	n.a.	n.a.	n.a.	n.a.	2/2	8/10	2/4	
GLDH (increased)	17	n.a.	n.a.	3/4	n.a.	n.a.	*	0/17	n.a.	4/31	n.a.	n.a.	n.a.	8/10	2/4	
Glucose (decreased)	11	4/9	6/6	1/3	1/1	n.a.	n.a.	n.a.	n.a.	n.a.	n.a.	n.a.	n.a.	n.a.	n.a.	
Ammonia (increased)	11	6/6	5/5	n.a.	n.a.	n.a.	n.a.	n.a.	n.a.	n.a.	n.a.	n.a.	n.a.	n.a.	n.a.	
ALP (increased)	10	n.a.	n.a.	2/3	1/1	n.a.	n.a.	n.a.	n.a.	7/31	n.a.	n.a.	n.a.	n.a.	n.a.	
*Liver enzymes not specified* *(* *3/4 AST/GGT/SDH/GLDH* *increased)*	*8*						8/15*									

Abbreviations: ALP, alkaline phosphatase; AST, aspartate aminotransferase; EqPV‐H, equine parvovirus hepatitis; GGT, gamma‐glutamyl transferase; GLDH, glutamate dehydrogenase; n.a., data not available; SDH, sorbitol dehydrogenase.

To investigate the disease association of the virus, horses have been experimentally infected using EqPV‐H‐contaminated equine serum or biological products.^16^, ^21^ Experimental infection resulted in subclinical hepatitis, demonstrated by increased liver enzymes, without clinical signs in most cases.^16^, ^21^ One experimentally infected horse developed signs of hepatitis, including icterus, mild lethargy, and inappetence of 6 days' duration.^16^, ^21^ In all horses, peak viraemia was observed approximately 5 weeks after infection, with the highest viral load detected in the serum and liver.^21^ Furthermore, viral DNA was also present in various organs and body fluids, such as synovial fluid, CSF, heart, kidney, colon, jejunum, synovium, salivary gland, bone marrow, lymph node, spleen, spinal cord, and lung.^21^ The virus persisted at low levels in the majority of tested body fluids and tissues for at least 15 weeks after infection.^21^ In the livers of experimentally and naturally infected horses, EqPV‐H replication was demonstrated in hepatocytes by in situ hybridisation (ISH). This was associated with hepatocyte necrosis and infiltration of lymphocytes.^21^, ^26^ The lymphocytic infiltrates in the periportal areas could be indicative of an acute immune reaction, possibly involving cytotoxic lymphocytes directed towards the virus‐laden hepatocytes.^16^, ^21^, ^42^ These findings provide evidence of viral hepatotropism, and the significance of EqPV‐H in other organs and body fluids should be investigated in future studies.

In experimentally infected horses, viraemia developed 0‐6 weeks after inoculation, followed by a close temporal association between peak viraemia, seroconversion, and the onset of hepatitis approximately 5‐8 weeks after infection.^21^ This evidence supports the hypothesis of EqPV‐H as a cause of Theiler's disease, with case histories confirming a 4‐ to 10‐week incubation period prior to clinical disease. Although the viraemia decreased after acute hepatitis, most experimentally infected horses remained viraemic for several weeks, and viral DNA remained detectable in different organs for at least 15 weeks after infection.^16^, ^21^ In nonexperimental cases of Theiler's disease, the virus remained detectable in survivors for 13‐20 months, indicating viral persistence.^14^, ^27^ Similarly, parvoviruses infecting other species (eg, the human parvovirus B19) can persist in serum and tissues for months to years.^43^, ^44^, ^45^ Longitudinal dynamics of viral load in serum and tissues of a large number of horses remain to be investigated. Immunological responses underlying viral persistence versus viral clearance and clinical versus subclinical infections also warrant future research.

Although infection studies have provided strong evidence of the association between EqPV‐H infection and acute hepatitis or Theiler's disease in horses,^16^, ^21^ other contributing factors cannot currently be excluded. Horses have been infected with EqPV‐H‐positive equine tetanus antitoxin^16^ or serum.^21^ Following similar infection studies with TDAV, it later became apparent that horses were, in fact, coinfected with EqPV‐H.^13^, ^16^ Therefore, it is imperative that existing evidence be evaluated critically. As a purified EqPV‐H inoculum is still lacking and would be essential to prove hepatitis causation, this should be considered a future research priority.^2^ Although attempts to propagate the virus in cell culture have been unsuccessful to date,^16^ this would enhance the understanding of EqPV‐H pathogenesis, and efforts should be made to address this area of research.

## 6 TRANSMISSION

### 6.1 Iatrogenic horizontal transmission

Experimental EqPV‐H infection of horses has been achieved by parenteral inoculation with virus‐containing tetanus antitoxin, serum, and, most recently, allogeneic mesenchymal stromal cell preparations.^16^, ^21^ Investigation of commercial equine serum pools, intended for manufacturing serum‐based products for medical and research purposes, revealed EqPV‐H DNA in 11/18 samples, with a viral load up to 10^5^ copies/ml.^37^ Iatrogenic transmission of EqPV‐H has been implicated in multiple cases of equine serum hepatitis, following administration of commercial equine plasma, allogenic stem cells, and, most commonly, tetanus antitoxin.^14^, ^16^, ^21^, ^26^ It has been suggested that the frequently observed association between serum hepatitis and tetanus antitoxin administration could be attributed to the widespread use of tetanus antitoxin in horses, as well as the fact that it is produced as a pooled donor product.^14^ Pooling of donor serum potentially increases the risk of virus contamination, compared with products like plasma and allogenic stem cell inoculations originating from single donors.^14^

### 6.2 Noniatrogenic horizontal transmission

Hepatitis, ranging in severity from subclinical to fatal, has also been reported in EqPV‐H‐positive horses with no recent history of biological product administration and in horses that were only in contact with recipients of biological products.^15^, ^25^, ^32^, ^36^ EqPV‐H infection in the absence of biological product administration and frequent detection in in‐contact horses highlight the infectious and potentially contagious nature of this virus and the need for further investigation into natural routes of horizontal transmission.^15^, ^25^ Considering the confirmed transmission of EqPV‐H through blood products, the hypothesis of arthropod vector‐borne transmission remains plausible.^15^, ^21^ EqPV‐H transmission by horse flies could, however, not be demonstrated in a recent study and was hypothesised to require a large number of bites.^21^ Intermittent shedding of EqPV‐H via the nasal, oral, and faecal routes was recently reported for experimentally infected horses.^21^ Although shedding events were centred around the time of peak viraemia, it could continue for 10 weeks after infection.^21^ Additionally, the oral route of infection was confirmed in one horse inoculated orally with EqPV‐H‐positive serum.^21^ Infection via intranasal inoculation with EqPV‐H‐positive serum was unsuccessful.^21^

### 6.3 Vertical transmission

Investigations have not yielded any evidence of viral transmission in utero.^21^, ^35^ However, 19/24 foals that were EqPV‐H‐negative at birth became infected within their first 7‐10 months of life.^21^ Horizontal transmission from EqPV‐H‐infected dams and/or EqPV‐H‐contaminated hyperimmune anti‐*Rhodococcus equi* plasma administered to foals was identified as possible sources of infection in these foals.^21^

### 6.4 Cross‐species transmission

Unlike EqHV—for which detection of antibodies and nucleic acid in samples from donkeys confirmed cross‐species transmission^46^—there is currently no evidence of EqPV‐H transmission to other species of equines.^33^ Although recent studies shed more light on the range of transmission possibilities,^21^ further investigation is warranted. The risk of viral transmission by subclinically infected horses and prophylactic hygiene measures is of particular importance. Based on the evidence supporting iatrogenic transmission via contaminated equine‐origin biological products, testing of such products during manufacturing and prior to distribution is strongly recommended. In 2019, the US Department of Agriculture (USDA) implemented regulations pertaining to EqPV‐H testing of donor horses, as well as EqPV‐H testing and the labelling of antitoxin, antibody, serum, or plasma products of equine origin.

## 7 RISK FACTORS: AGE, BREEDING, AND SEASON

The vast majority of EqPV‐H surveillance has been performed in adult horse populations. A slight increase in the fraction of seropositive horses was observed in association with an increasing number of years in breeding.^35^ However, it was speculated that this association could be attributed to the advanced age of the horses.^35^ Increasing age as a risk factor for EqPV‐H infection was further supported by the findings of a surveillance study in Austrian horses. In this study, the highest infection prevalence was found in 16‐ to 31‐year‐old horses.^33^ A recent study by Tomlinson et al reported EqPV‐H in foals for the first time, with 79% (19/24) of foals that were EqPV‐H PCR‐negative at birth, becoming PCR‐positive within their first year of life.^21^ As Theiler's disease has not previously been reported in this age group, it was suggested that the lack of susceptibility to severe hepatic necrosis in foals is due to other factors, such as immunologic development, rather than a lack of susceptibility to infections.^21^

Naturally occurring cases of EqPV‐H‐associated Theiler's disease, with no history of recent administration of biological products, have primarily been reported during the late summer and autumn—from July to October.^15^, ^25^, ^26^ Based on this apparent seasonal occurrence, vector‐borne transmission of EqPV‐H must still be considered likely in Theiler's disease cases not associated with biological product administration.^21^

## 8 PRACTICAL AND CLINICAL RELEVANCE

Despite EqPV‐H research still being in its infancy, the clinical relevance of this virus is becoming increasingly apparent. Awareness of the potentially fatal consequences and legal implications of administration of contaminated biological products is crucial for equine practitioners. EqPV‐H is an important differential diagnosis that has to be considered and investigated in cases of clinical and subclinical equine hepatitis—even in the absence of a history of biological product administration. In future, a better understanding of transmission routes of the virus will enable practitioners to advise owners on the implementation of appropriate preventative protocols. Although the potential detrimental effect of EqPV‐H infection—particularly persistent, subclinical infections—on athletic performance of horses remains to be thoroughly investigated, this does warrant consideration in equine athletes.

## CONFLICT OF INTERESTS

No competing interests have been declared.

## AUTHOR CONTRIBUTIONS

Review conception was done by AS Ramsauer, M. Badenhorst, and JMV Cavalleri. The review was primary written by AS Ramsauer and M. Badenhorst, and overall supervision was provided by JMV Cavalleri. All authors commented and discussed the drafts and approved the final version.

### PEER REVIEW

The peer review history for this article is available at https://publons.com/publon/10.1111/evj.13477.
